# Shifting the narrative: from “the missing men” to “we are missing the men”

**DOI:** 10.1002/jia2.25526

**Published:** 2020-06-26

**Authors:** Anna Grimsrud, Wole Ameyan, James Ayieko, Tanya Shewchuk

**Affiliations:** ^1^ HIV Programmes and Advocacy Department International AIDS Society Cape Town South Africa; ^2^ Global HIV, Hepatitis and Sexually Transmitted Infections Programmes World Health Organization Geneva Switzerland; ^3^ Research Care and Training Program Kenya Medical Research Institute Nairobi Kenya; ^4^ Global Delivery, Bill and Melinda Gates Foundation Seattle WA USA

**Keywords:** men, HIV, differentiated service delivery, 90‐90‐90, testing

Thirty years into the HIV response, there is growing recognition that engaging men will be key to reaching the global UNAIDS fast‐track targets of 90‐90‐90 by the end of 2020 – whereby 90% of people living with HIV know their status, 90% of those who know they are positive are on antiretroviral therapy (ART) and 90% of those on ART are virally suppressed [1]. The most recent global HIV data through 2018 show that progress towards the 90‐90‐90 targets for men lags behind at 75‐74‐85 compared to 84‐81‐87 for women [2]. Looking at the second 90, ART coverage is considerably lower for men than women globally (68% vs. 55%), and consistently lower in all of the seven World Health Organization (WHO) regions except Latin America [3].

Since the 2017 UNAIDS publication of “Blind spot: Reaching out to men and boys,” the global trend of poorer outcomes across the HIV care cascade for men has gained traction and focus from PEPFAR programmes, national departments of health, implementing partners and global normative agencies [4, 5, 6, 7, 8]. Furthermore, recent guidance from the WHO does highlight gender differences in HIV outcomes, including the substantial gap in reaching men with HIV testing services [9].

Our call for abstracts for this supplement highlighted this growing attention and sought to collect and promote approaches to reaching men with HIV testing, prevention, treatment, care and support services. The interest in this topic was reflected in the over 100 abstracts that we received, which emphasized several salient points on where the gaps are and where we should be headed.

First and foremost, it is time to shift away from a narrative that looks at men from a “safe” distance, blames men for poor health‐seeking behaviour and focuses on men solely to improve the health of their partners and children. Men need and are willing and deserve to have access to services for their own health. Engaging men in health services for their own health can further provide an entry point for programmes that may have a positive impact on improving the health of their families and communities.

Our second takeaway is that the current system is not working for anyone – all populations are negatively impacted by the current gender norms. As highlighted by The Lancet in their recent series on gender, equality, norms and health, “rigid gender norms undermine the health and wellbeing of all people – girls and women, boys and men, and gender minorities” [10]. This is true in HIV where, as outlined, men are not accessing and benefiting from ART in the same way as women, while at the same time, incidence rates among adolescent years girls and young women remain unjustifiably high [11] and outcomes among key populations, including men who have sex with men and transgender people, remain disturbingly poor [3].

Thirdly, given the global HIV response and the current spotlight on men, HIV programmes may be uniquely positioned to drive a larger men’s health agenda and plans within countries that are adapted to different settings. Data highlighting worse outcomes for men compared to women are not unique to HIV. The global burden of disease data sheds light on mortality rates presenting sex‐disaggregated data across geographies and notes that improvement is less pronounced, particularly for adult males where in several countries progress in mortality was “stagnant or increasing” [12]. There is evidence of increased morbidity among men from infectious diseases including tuberculosis (TB) [13, 14] and other conditions including cardiovascular diseases, respiratory diseases and injuries [15]. Higher rates of co‐infection with TB were demonstrated by Osler *et al*. in this supplement where men living with HIV were twice as likely to have TB compared to women living with HIV [16]. The experiences of the “Khotla” male‐centred services in Lesotho highlight both the importance of having a physical space within the health system for men and that a non‐vertical, comprehensive men’s health services offering is appealing to men [17].

Despite the large number of abstracts reviewed for this supplement, there were limited data and evidence to show what works. Much of the data describes challenges or current pilot programmes (often focused on HIV testing), with few examples highlighting where men’s health has been mainstreamed and health systems have been responsive to their needs.

Despite this, there is a strong case for health systems that are people‐centred and can be sensitive and responsive to the attributes of clients [18]. Put differently, we need differentiated service delivery for different populations. There are increasing calls for “x‐friendly services” which all include integration, service hours that work for patients, are offered by educated and sensitive staff and involve peers. This is universal – for men, for adolescents, for key populations, for women – the bottom line is that people, including men, want and need services that respond to their needs. The balance is to ensure these services can be offered within a public health approach and in resource‐constrained settings.

In this supplement, four key themes emerged. First, health systems are structurally gendered to address women’s health needs. Second, while there are considerable efforts, including through the MenStar Coalition [19], to reach younger men, there is a large number of “older” men (those over 35 years old) who require HIV services. The third theme is that programmes are going to need to be more creative and strategic to access and test men who truly do not know their HIV status. Some of the interventions studied around testing may be retesting those who know their status instead of reaching men who are unaware of their HIV‐positive status. This insight underscores not only the need for testing interventions to reach those unaware of their status but also corresponding services adapted so men start and stay on treatment. Finally, as described above, it’s time for a narrative shift away from “men as the problem” to one that views men as a group that is interested in health and able to be part of the solution where health systems adapt to meet men where they are.

## 1 RECOGNITION AND ENGAGEMENT OF MEN BY HEALTH SYSTEMS

In many resource‐limited settings, health systems are largely designed to address critical maternal and child health needs. This point is described in detail by Dovel *et al.* in relation to Malawi [20] and complements earlier work highlighting that during adulthood, women (excluding pregnant women and those with children under two years of age) have 19 hours per year of interaction with the health system compared to just three for men [21]. The gendered health services are further exacerbated by the reality that in many high HIV prevalence countries, women are the majority of the health workforce. Men are therefore absent from the health system both as patients and as providers. As a start, programming and global guidance on HIV should include and recognize men as a critical group that requires HIV prevention, care and treatment. Engagement and involvement of men, by health systems, primarily as a group that is interested in their own health is a reasonable first step in addressing the glaring gaps. The “men gap” needs to be closed to achieve the 90‐90‐90 global targets by 2020 and to end the HIV epidemic by achieving 95‐95‐95 by 2030.

## 2 HIV SERVICES ARE NEEDED FOR OLDER MEN

Historically, efforts to reach men with services has been done to ensure services for their partners. As such, there are considerable efforts to reach men with HIV programming, reduce HIV infections among their partners and/or improve outcomes for their partners who are living with HIV. However, when looking at HIV population pyramids it is clear that the largest numbers of HIV‐positive men currently not engaged in services are among those over 35 years of age, which aligns with the age bands of highest prevalence. In the work by Gottert *et al.*, 20% of the sample was “older high risk groups” [22] highlighting the critical need for services focussed on older men. It is also critical to reach these men sooner, before they are acutely ill. In data from Western Cape province in South Africa, 39% of the men first presented with a CD4 count below 200 copies/mL, had increased probability of death compared to women and were less likely to start ART compared to women [16]. The lowest uptake of ART was among men not co‐infected TB, 26% less likely than men coinfected with TB.

## 3 ENHANCING HIV TESTING TO REACH THOSE WHO HAVE NOT BEEN REACHED

The advent of HIV self‐testing (HIVST) has accelerated interest and modalities to reach men with HIV testing [23]. Data from the Kwa‐Zulu Natal province in South Africa in this supplement highlights how both oral and blood‐based HIVST are reaching men [24]. In the study by Barnabas *et al.*, HIVST reached a high number of men with the majority having a suppressed baseline viral load suggesting that there are unanswered questions on retesting behaviours as they were already active on treatment and knew their HIV status [25]. In Zambia’s Community Impact to Reach Key and Underserved Individuals for Treatment and Support (CIRKUITS) project, the index testing approach was successful in reaching both a high volume and yield of HIV‐positive men [26]. It is critical to interrogate this data, given than 75% of the traced contacts had an unknown HIV status. By comparison, Zambia’s populationȐbased HIV impact assessment data from 2016 estimated the first 90 gap among men to be only 29% [27]. Therefore, it is important to consider the proportion of men in the CIRKUITS project not disclosing their HIV status to the CIRKTUS staff. HIVST is a critical intervention to reach those who would not otherwise test, and the evidence of this is further bolstered by the findings of Napierala *et al*. In this study, women at increased vulnerability to HIV did secondary distribution of HIV self‐test kits – each woman gave HIVST to a median of three partners with 94% offering a kit to their primary partner [28].

## 4 REFRAMING THE NARRATIVE

As outlined above and stated in the viewpoint of Makusha *et al.* [29], now is a critical moment to change the discussion and acknowledge the unique and underserved health needs of men. We call on national ministries of health to consider a “men’s health agenda” and how to ensure the health system is inclusive of men. Importantly, the narrative needs to consider men in their diversity and across the life course. Bhattacharjee *et al.* [30] described the HIV cascade among a sample of Kenyan men who have sex with men, comparing those who use physical sites, physical and virtual sites and virtual sites to meet sex partners. On life course, there needs to be a recognition that the harmful norms and behaviours that drive negative health outcomes among men are likely to be internalized during adolescence. Health systems need to address the needs of adolescent boys with a view to harnessing the triple dividend of benefits for adolescents now, for their future adult lives, and for the next generation [31].

In conclusion, at this juncture the HIV response should support HIV programming for men from prevention to testing, care and treatment by considering their needs. To improve services, it is essential that health services acknowledge that we have been missing men, and that it is our collective responsibility for health systems to be people‐centred to address the needs of all people.

## COMPETING INTERESTS

None of the authors have competing interests.

## AUTHORS’ CONTRIBUTIONS

AG wrote the first draft. WA, JA and TS authors provided comments and input. All authors approved the final submission.
